# The fear that remains: Associations between trauma, related psychopathology, and fear-potentiated startle in youth resettled as refugees

**DOI:** 10.1002/dev.22385

**Published:** 2023-05

**Authors:** Lana Ruvolo Grasser, Bassem Saad, Celine Bazzi, Hiba Abu Suhaiban, Dalia F. Mammo, Ragda Izar, Noor Abou Rass, Sterling J. Winters, Raya Nashef, Ayat Abed Ali, Arash Javanbakht, Tanja Jovanovic

**Affiliations:** Department of Psychiatry and Behavioral Neurosciences, Wayne State University School of Medicine, Detroit, Michigan

**Keywords:** biomarkers, fear extinction, fear-potentiated startle, PTSD, refugees, trauma

## Abstract

Fear-potentiated startle (FPS) can be used to measure fear and safety learning—behaviors affected by trauma that may map onto posttraumatic stress disorder (PTSD). Therefore, FPS could be a candidate biomarker of trauma-related psychopathology and a potential identifier of trauma-exposed youth in need of focused treatment. We enrolled *n* = 71 (35 females, *M*_age_ = 12.7 years) Syrian youth exposed to civilian war trauma. Eyeblink electromyogram (EMG) data from a differential conditioning FPS paradigm were obtained 2.5 years after resettlement. Youth provided self-report of trauma exposure (Harvard Trauma Questionnaire) and PTSD symptoms (UCLA PTSD Reaction Index). While FPS during conditioning was not associated with symptoms, associations with psychopathology emerged in fear extinction. Probable PTSD was associated with FPS in the last block of extinction, such that FPS to threat cue was significantly greater in the PTSD+ group compared to the PTSD− group at the end of extinction (*F* = 6.25, *p* = .015). As with adults, we observed a deficit in extinction learning but not fear conditioning in youth with PTSD. These results support the use of trauma-informed cognitive behavioral therapy based on the learning principles of extinction in youth with PTSD.

## 1 | INTRODUCTION

One in five youth in the United States are exposed to trauma (Finkelhor et al., 2009). Some populations are exposed to trauma at higher rates, and youth who flee home countries represent one such group (Yayan et al., 2019). While fear and safety learning are critical for survival (Fanselow, 1994; Lohr et al., 2007) for a subset of youth exposed to trauma, these initially adaptive physiological and psychological responses can transition to pathological or maladaptive. As a result of trauma exposure, an estimated 5% of adolescents develop posttraumatic stress disorder (PTSD). For children living in war-torn countries, this prevalence may be higher (for example, 9–54% in refugee youth; Javanbakht et al., 2018; Perreira & Ornelas, 2013; Solberg et al., 2020), and higher rates of trauma experienced by many refugees may put individuals at greater risk for chronic PTSD (Michopoulos et al., 2019). The physiological responses that underlie detection of threat and engagement of defensive behaviors (Fanselow, 2018) likely contribute to long-term negative health consequences when such responses are chronically mounted even in the context of safety. Fear and safety learning are quantifiable in laboratory and clinical settings and may provide predictors of risk and potential treatment targets for fear and anxiety-related psychopathology in children and adolescents (Giedd, 2004; Gogtay et al., 2004).

Fear-potentiated startle (FPS) combines Pavlovian fear conditioning and the acoustic startle response (ASR) to measure fear and extinction learning (Davis et al., 1993; Jovanovic et al., 2005). Fear potentiation of the ASR can be modulated through associative learning procedures (Koch, 1999). The fear response component is measured as the increased magnitude of the startle response to the acoustic startle probe when measured during presentation of a conditioned stimulus (CS+) that predicts an aversive event/stimulus [unconditioned stimulus (US); “threat”], compared to presentation of the startle probe alone (Grillon & Morgan, 1999; Morgan et al., 1995) and the presentation of the startle probe with another conditioned stimulus (CS−) that is never paired with the aversive US. The ability to discriminate between threat and safety cues can be measured as the difference in the magnitude of the startle response to the safety cue (CS−) subtracted from the magnitude of the startle response to the threat cue (CS+).

Following fear conditioning, extinction learning can facilitate the formation of a new, competing memory that indicates the absence of the previously conditioned threat (Myers & Davis, 2002). The persistence of heightened FPS response to previously conditioned threat cues after extinction learning is considered a candidate biomarker for PTSD (Grasser & Jovanovic, 2021; Maples-Keller et al., 2022; Michopoulos et al., 2015; Milad et al., 2009; Norrholm et al., 2011). Deficits in extinction of FPS responses have been repeatedly observed in adults with PTSD (Maples-Keller et al., 2022; Norrholm et al., 2011); for example adults with PTSD compared to trauma-exposed controls have shown elevated FPS responses to the CS+ during early and middle stages of extinction, representing a higher “fear load” to be extinguished (Norrholm et al., 2011). Additionally, individuals with more severe reexperiencing PTSD symptoms had greater FPS to CS+ compared to those with less severe reexperiencing symptoms (Norrholm et al., 2011). Reexperiencing symptoms may reflect individuals’ weakened ability to inhibit fear (Amstadter et al., 2009; Norrholm & Jovanovic, 2010; Rothbaum & Davis, 2003). Promisingly, psychotherapeutic treatment of PTSD can improve extinction learning and retention (Maples-Keller et al., 2022). FPS has also been used to study anxiety and anxiety-like behaviors in youth; however few studies have used FPS to study psychopathology and posttraumatic stress in youth (Gamwell et al., 2015).

Trauma may impact development of fear and extinction learning, neural networks related to threat and safety processing, and be associated with psychopathology. While some studies have investigated differences in extinction learning among youth exposed to trauma, youth with anxiety disorders, and healthy youth, none have specifically looked at extinction learning in youth with PTSD nor has FPS more specifically been broadly examined within trauma-exposed pediatric cohorts. Syrian refugee youth represent a widely understudied population which merits investigation (Grasser, 2022). Identifying potential biomarkers of trauma-related symptoms may be especially important when working with immigrant and refugee communities (Javanbakht & Grasser, 2022), where verbal presentation of symptoms may differ based on cultural norms and a potential language barrier. In the present study, we aimed to measure FPS to threat and safety cues in youth endorsing posttraumatic stress symptoms across a continuum. At the end of fear conditioning, we hypothesized that FPS would be associated with severity of posttraumatic stress symptoms. We also hypothesized that youth with PTSD would show deficits in extinction of learned fear compared to those without PTSD, with the goal of testing whether the same patterns of fear learning and extinction that have been observed in adults with PTSD emerged in youth with PTSD.

## 2 | METHODS AND MATERIALS

**FIGURE 1:**
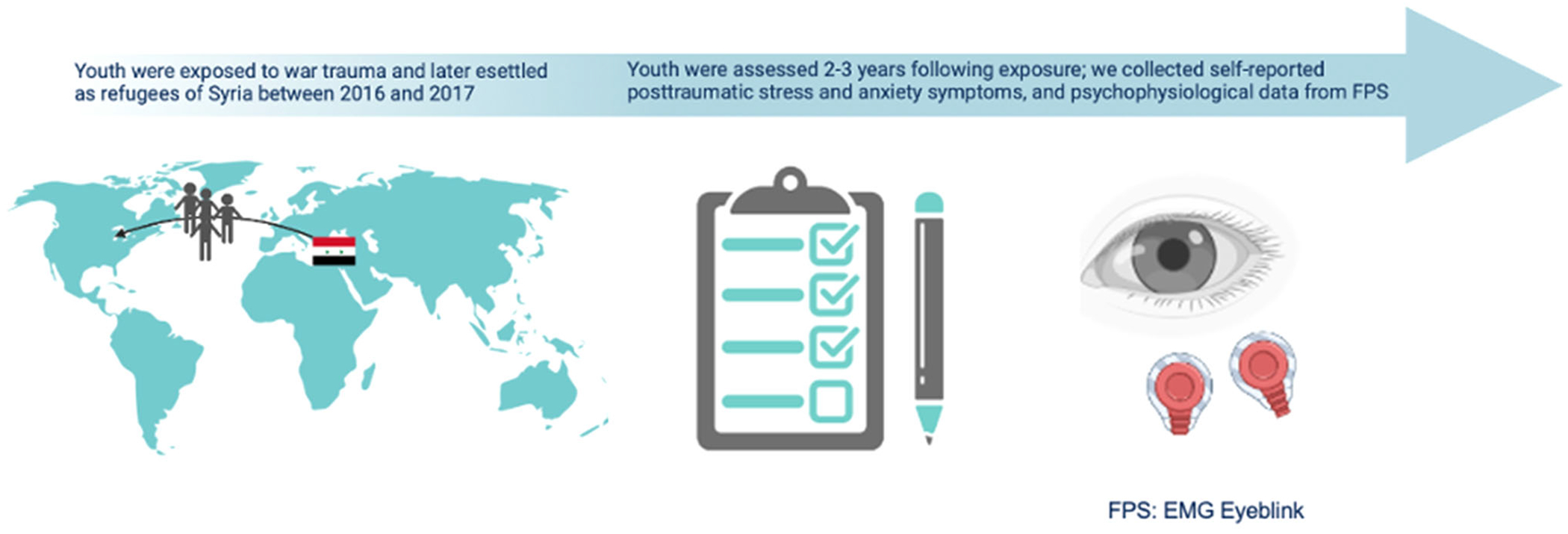
Study timeline. Youth resettled as refugees of Syria (*n* = 71; 35 females) in the United States between 2016 and 2017 participated in a fear potentiated startle paradigm (between 2019 and 2021) during which electromyogram (EMG) was used to record eyeblink startle response during the task. We then examined FPS to threat and safety cues during fear learning and extinction, and associations with trauma exposure and related psychopathology. Figure created in Biorender.

A summary of study methods is depicted in Figure 1.

### 2.1 | Participants

Participants for this study were recruited from a larger study of refugee health previously described (Javanbakht et al., 2018; Javanbakht et al., 2021). These youth resettled in the United States between 2016 and 2017, and participated in the present study between 2019 and 2021. Inclusion criteria were (1) aged 7–17, (2) resettled as a refugee of Syria between 2016 and 2017, and (3) willing and able to give oral assent (ages 7–12) or written assent (ages 13–17) with a parent or legal guardian willing and able to give written parental permission/informed consent. Exclusion criteria were (1) being a ward of the court, (2) current diagnosis of autism or psychosis spectrum disorders, and (3) inability or unwillingness to provide assent or consent. The materials and methods described herein were approved by the Institutional Review Board (IRB) at Wayne State University (IRB #012416B3F).

Sample size was determined using average startle responses to CS+ for youth with clinically significant anxiety symptoms and those with subthreshold symptoms *(M* = 85.61 and *M* = 26.17, respectively) from a previous study (Jovanovic et al., 2014). PTSD data were unavailable. Using these data, we determined that a sample size of 70 was sufficient to detect moderate effects of group with an alpha level of .05 and 85.7% power. Seventy-one individuals were enrolled and completed the protocol for the present study.

### 2.2 | Questionnaires

All self-report assessment measures were administered by bilingual clinicians and were available in both English and Arabic. Questionnaires were translated from English to Arabic by a native speaker, back-translated to English by a separate Arabic speaker to ensure consistency and accuracy in translations, and finally certified and approved by external reviews.

Trauma exposures were assessed using the Harvard Trauma Questionnaire (HTQ), which queries traumatic events more specific to refugee and non-Western cohorts (Mollica et al., 1992; Rasmussen et al., 2015). A modified version of the HTQ included 21 items from the trauma screening inventory section, to which participants would respond “yes”—indicating they had experienced that event and coded as a “1” and without distinction regarding the number of times the individual had experienced that event—or “no”—indicating they had not experienced that event and coded as a “0.” The item responses were then summed to provide an indication of the number of unique traumatic events youth had been exposed to, with a possible range of 0 to 21. Cronbach’s alpha in this sample was 0.872.

Severity of posttraumatic stress was assessed using the UCLA PTSD Reaction Index for Children and Adolescents (UCLA-PTSD-RI) for DSM 5. The UCLA-PTSD-RI is a 31-item questionnaire on which participants indicate how much they have been bothered by particular symptoms within the last month, ranging from none of the time (0) to most of the time (4). A total score of 35 or higher is indicative of probable PTSD (Kaplow et al., 2020). The UCLA PTSD-RI has been recommended for use with refugee populations, including those with non-Western backgrounds (Miller et al., 2019), and has been found to be reliable in Middle Eastern/North African (MENA) refugee populations (Grasser et al., 2021; Javanbakht et al., 2018). Cronbach’s alpha in this sample for the full measure was .936.

All questionnaires were scored according to published guidelines corresponding to each instrument.

### 2.3 | Fear-potentiated startle paradigm

**FIGURE 2:**
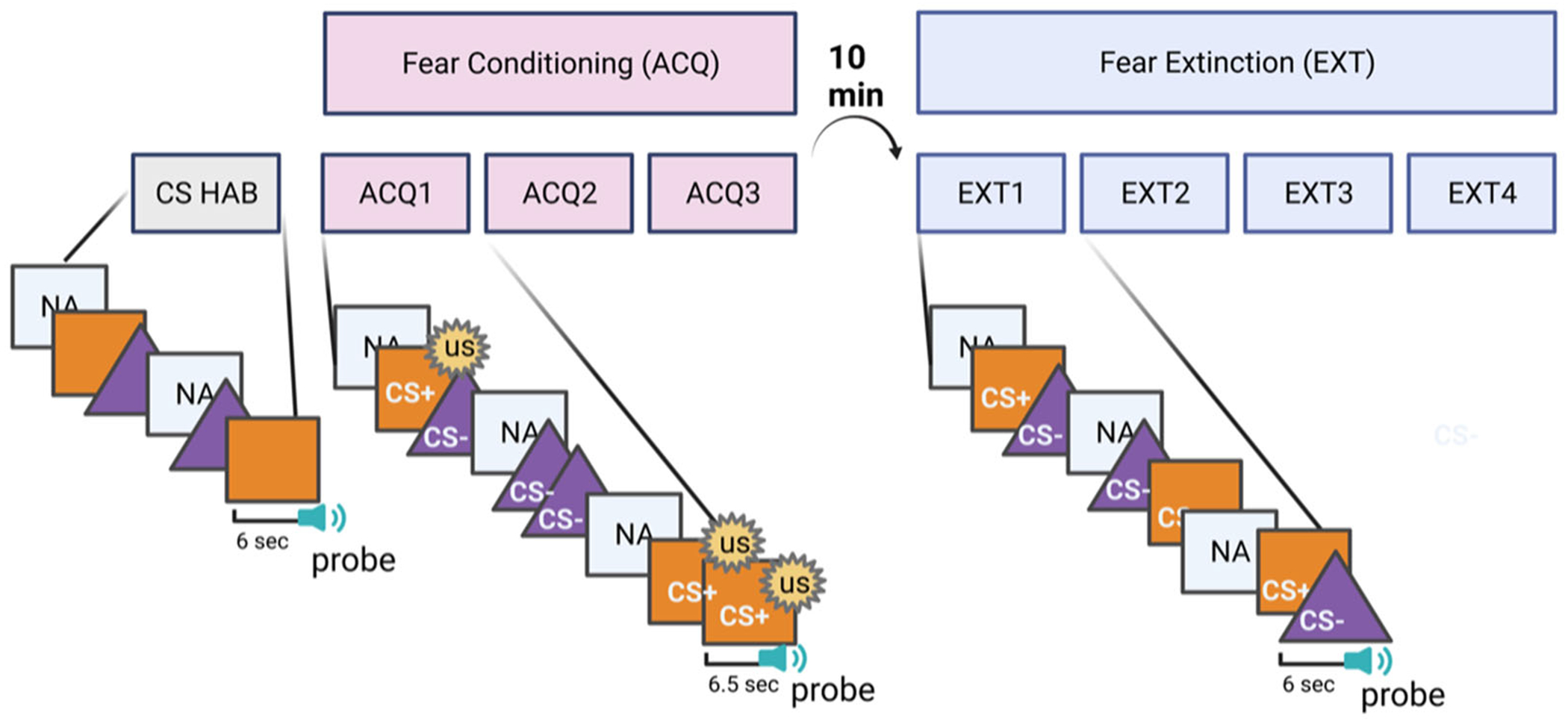
Fear-potentiated startle experimental paradigm. During habituation (CS HAB), each of the conditioned stimuli (orange square and purple triangle) is presented for 6 s, coterminating with the 500 ms acoustic startle probe. The noise alone (NA) is also presented. During fear conditioning, the 100 ms air blast (unconditioned stimulus; US) is paired with the CS+ (orange square), coterminating with the CS+ and NA. The CS− (purple triangle) is never paired with the US. During fear extinction, the CS+ is not presented with the US such that a new, competing memory is formed signaling the CS+ is now safe.

Biopac MP160 for Windows (Biopac Systems, Inc.) was used to collect electromyogram (EMG) data during the startle protocol. Experimental stimuli were presented using SuperLab 5.0 for Windows (Cedrus, Inc.) and synchronized with psychophysiological data collection. The fear-potentiated startle experimental design used in this study is a widely used paradigm that has been validated and replicated in numerous adult and youth studies (Figure 2) (Jovanovic et al., 2005; Jovanovic et al., 2006; Reeb-Sutherland et al., 2009; Stenson et al., 2021). The conditioned stimuli (CSs) were two different colored shapes (one triangle, one square, with color options of blue, purple, and orange) appearing on a computer monitor 3 feet in front of the participant. The computer monitor was also equipped with a webcam, for continuous monitoring of the participant during the task to ensure that the participant was awake, attentive, and not experiencing any significant distress or discomfort. Recordings of the participant during the task were not stored for future analyses. The startle probe was a 106 dB, 40 ms white noise burst delivered binaurally through headphones. Prior to the task, all participants underwent a hearing test to ensure they would be able to perceive the acoustic startle probe. The unconditioned stimulus (US) was a 100 ms, 80 p.s.i air blast directed at the larynx. During the fear-potentiated startle task, the acoustic startle probe is used in a Pavlovian conditioning paradigm to derive the eyeblink as the dependent variable, such that the magnitude of the startle response is expected to be greater when the auditory probe is presented during the CS+, compared to presentation of the CS− or noise alone (NA).

Prior to the conditioning phase, both CSs were presented, nonreinforced, in the habituation phase. During the habituation phase, the acoustic startle probe (NA), CS+ (without the US), and CS− trials were each presented two times. During conditioning, one CS was paired with the air blast (CS+) while the other CS was not paired with the air blast (CS−). CS+ was reinforced 100% of the time. The CS was presented for 6500 ms total; the CS+ was presented for 6000 ms prior to the onset of the startle probe, and coterminated with the US 500 ms after the presentation of the startle stimulus. The trial parameters for the CS− were similar to the CS+, with the startle probe delivered 6000 ms after stimulus onset; however, the US was never paired with the CS− (Figure 2). Conditioning consisted of three blocks, each with three CS+ trials, three CS− trials, and three noise alone trials (NA; no CS presented with the startle probe), for a total of 27 trials. In all phases of the experiment, inter-trial intervals (ITI) were randomized between 9 and 22 s. CS contingencies were counterbalanced across participants, that is, which CS (colored shape) was used as the CS+ and CS−. Participants rested for 10 minutes between fear conditioning and extinction. During extinction, the CS+ was never paired with the air blast. Extinction consisted of four blocks, each with three nonreinforced CS+ trials, three CS− trials, and three NA trials, for a total of 36 trials.

During the task, EMG was used to measure startle magnitude (eyeblink component) by placing two Ag/AgCl electrodes on the orbicularis oculi muscle: one 1 cm under the right pupil and the other lateral to the first electrode with a ground electrode placed on the mastoid bone behind the right ear. Data were sampled at 1 kHz. Acquired data were filtered and rectified in MindWare (MindWare Technologies, Inc. Gahanna, OH). High- and low-frequency cutoffs for EMG signal filtering were set at 500 and 28 Hz, respectively. Startle responses were assessed as the peak amplitude of the EMG contraction within a 20–200 ms window following the onset of the acoustic stimulus. Fear-potentiated startle responses were calculated as the magnitude of the startle response to threat (CS+) and safety (CS−) cues, adjusted for by subtracting startle responses to the acoustic probe alone (NA).

### 2.4 | Data analysis

Data were password protected and stored in REDCap (Harris et al., 2019; Harris et al., 2009), an electronic data capture tool hosted at Wayne State University. Data were exported to RStudio running R version 4.0.3 and SPSS version 27 for analyses. Data were screened for univariate outliers based on median absolute deviation, a method that is advantageous compared to other methods that are based on means and standard deviations, given that these statistics are influenced by outliers (Leys et al., 2013). 7.1% of values were identified as outliers and subsequently replaced with the nearest value. Little’s test (Roderick, 1988) was used to ensure that data were missing completely at random (MCAR), and nonsignificant chi-squared statistics indicated that data were MCAR, permitting imputation of missing data using multiple imputation. 8 iterations of multiple imputation were used for variables that had less than 50% of data MCAR.

Repeated measures ANOVAs (RMANOVAs) were used to determine whether there were significant effects of block, trial, and block by trial on FPS responses. Within and between-subjects effects of age, sex, trauma exposure, and posttraumatic stress were also tested in these models. When Mauchly’s test of sphericity was significant, the null hypothesis of equal variances was rejected, and appropriate corrections were adopted based on the epsilon statistic.

## 3 | RESULTS

### 3.1 | Demographics

Seventy-one (35F, *M*_age_ = 12.47, ±2.77 SD years) youth participated in the study. Twenty-eight were age 11 and under; and 43 were age 12 and older. Participants were all resettled from Syria and endorsed Middle Eastern/North African ethnicity as well as religion of Islam. Seven youth reported a medical condition and 4 reported medication use; however, none of the youth were being treated for psychiatric conditions. Youth endorsed an average of 4.66 (± 4.33 SD) unique traumatic events based on the HTQ. Twenty-three participants (32.4%) screened positive for probable PTSD based on a UCLA score of 35 or above; youth who screened positive for PTSD were exposed to significantly more unique traumatic events than those who did not screen positive, *t*(69) = 3.52, *p* <.001.

### 3.2 | Fear conditioning

**FIGURE 3:**
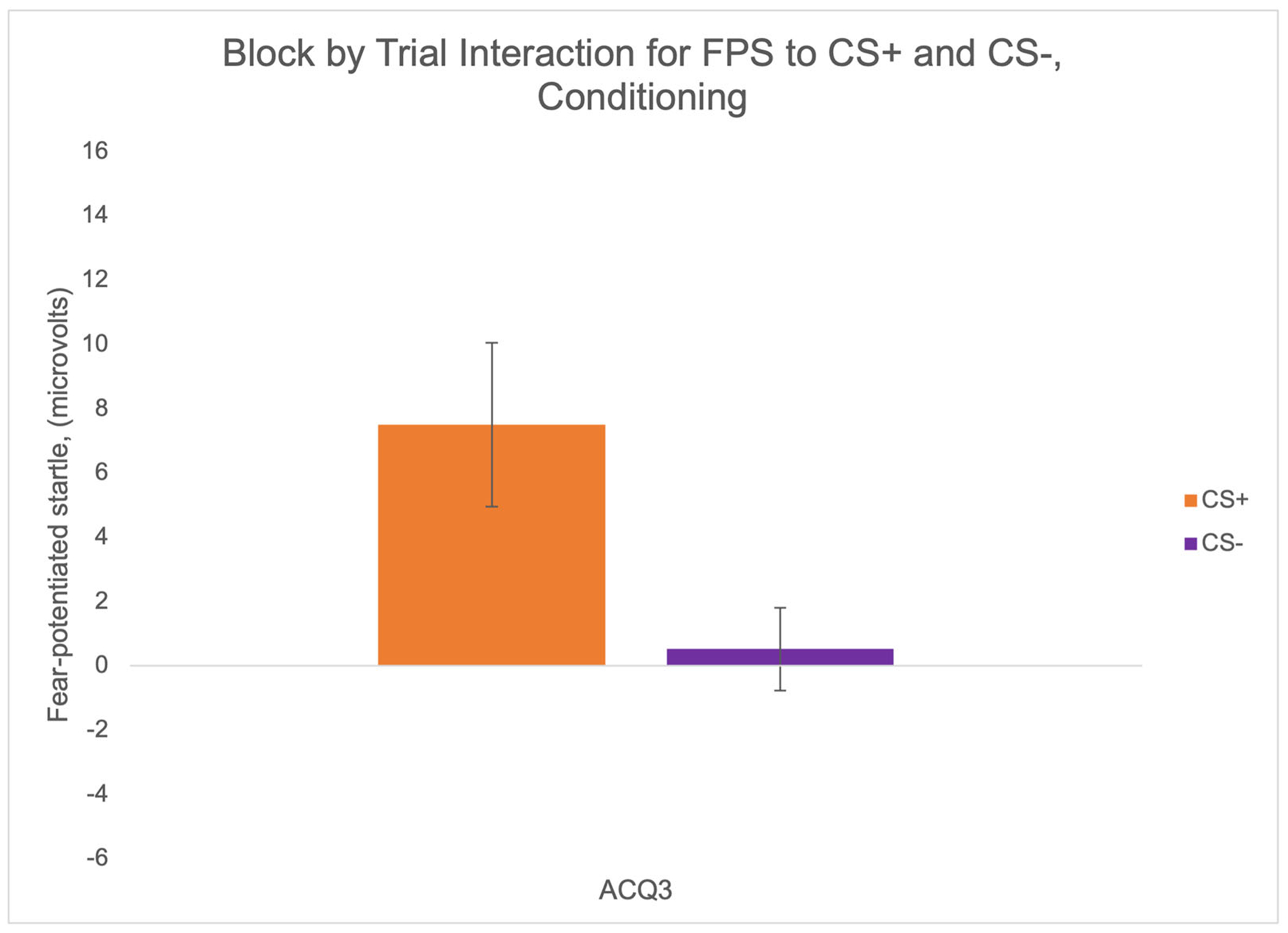
Evidence for successful fear conditioning. By the end of fear conditioning, EMG startle responses indicate fear potentiation and discrimination between threat (CS+, orange) and safety (CS−, purple) cues. Error bars reflect standard error of the mean.

A 4 × 3 RMANOVA was used to determine whether there were significant effects of block [habituation (HAB), acquisition block 1 (ACQ1), acquisition block 2 (ACQ2), and acquisition block 3 (ACQ3)], trial type [noise alone (NA), threat cue CS+, or safety cue CS−], and block by trial type. There was significant effect of trial type, *F*(1.85,129.64) = 7.05, *p* = .002, *η*_p_^2^ = .09, and a significant interaction effect of block by trial type, *F*(5.57,389.91) = 3.99, *p* < .001, *η*_p_^2^ = .05 (Figure 3). By the last block of fear conditioning (ACQ3), FPS was significantly greater to the CS+ than the CS−, *F*(1,70) = 9.34, *p* = .003, *η*_p_^2^ = .12, indicative of both fear potentiation and discrimination between threat and safety.

### 3.3 | Fear conditioning and trauma exposure

**FIGURE 4:**
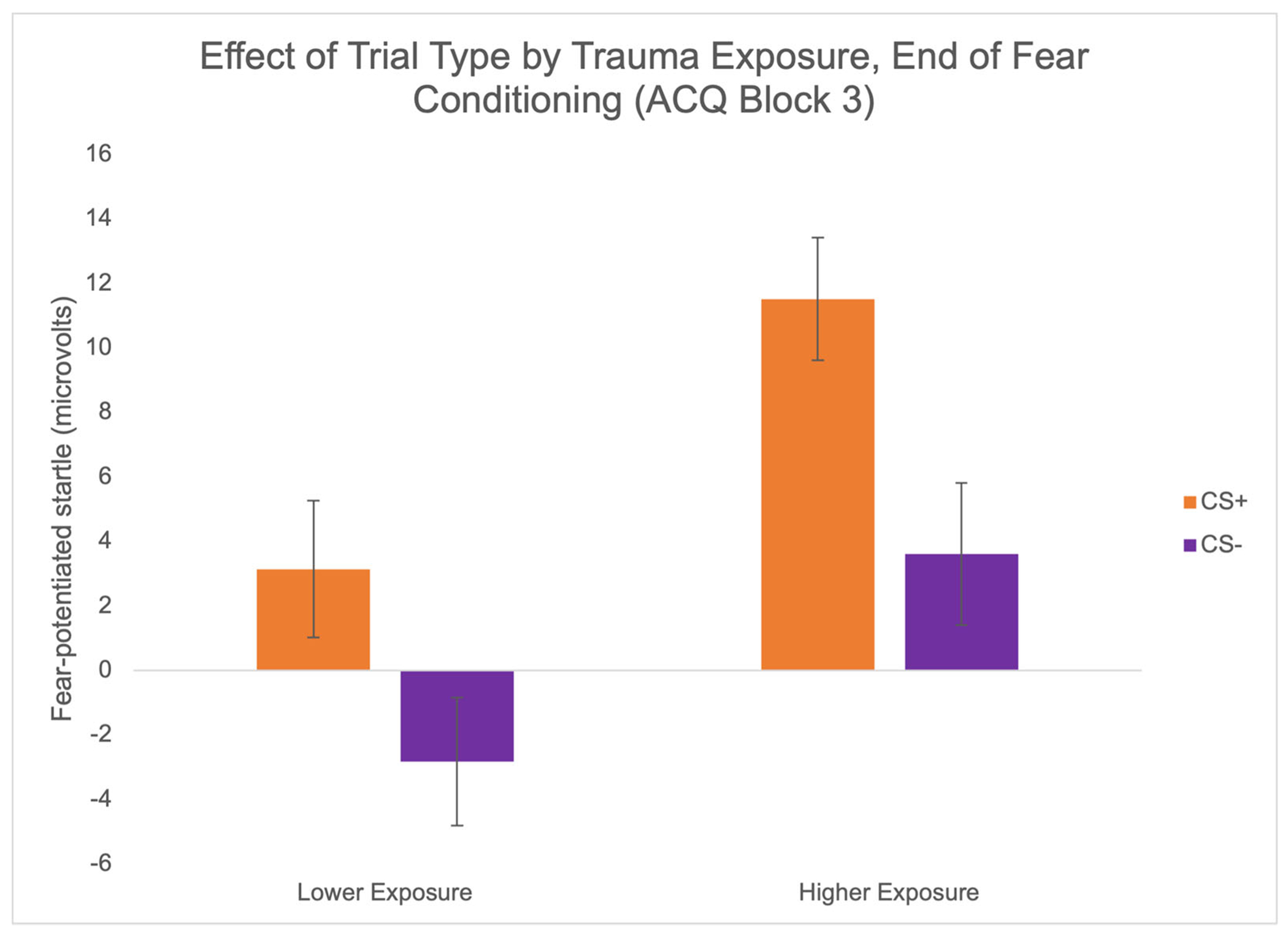
Trauma exposure is associated with fear conditioning. Significant between group effect of trauma exposure on FPS during last block of fear conditioning. Trauma exposure was based on median split; upon examination of the data, those categorized as “higher exposure” based on median split all had four or more traumas endorsed on the HTQ. Those categorized as “lower exposure” based on median split all had three or fewer traumas endorsed on the HTQ. Error bars reflect standard error of the mean.

We observed a significant between groups effect of trauma exposure when we categorized participants by severity (lower versus higher, based on median split) such that individuals with greater trauma exposure exhibited higher FPS compared to those with lower trauma exposure, *F*(1,69) = 5.27, *p* = .025, *η*_p_^2^ = .07 (Figure 4). Comparing trial type effects within each group, FPS during block 3 did not differ for CS+ compared to CS− in the lower exposure group, *F*(1,33) = 2.53, *p* = .121, *η*_p_^2^ = .07. However, FPS was significantly greater to the CS+ than the CS− in the higher exposure group, *F*(1,36) = 8.28, *p* = .007, *η*_p_^2^ = .19. Looking at trauma exposure as a continuous measure, we confirmed that youth with greater trauma exposure had higher FPS to both threat (*r* = .25, *p* = .035) and safety (*r* = .26, *p* = .032) cues at the end of conditioning.

Notably, the effects of trauma exposure on FPS to threat and safety cues were not significant when controlling for age, *F*(1,68) = 3.17, *p* =.080, *η*_p_^2^ =.05 (*M_age, lower exposure_* = 11.24, SD = 2.03; *M_age, higher exposure_* = 13.59, SD = 2.89). On average, the lower exposure group was significantly younger than the higher exposure group, *t*(69) = −3.95, *p* = .022, and age was significantly correlated with trauma exposure, *r*(69) = .52, *p* < .001.

There was no group (PTSD+/−) by trial interaction effect for the last block of acquisition (*F*(1,69) =.52, *p*=.472, *η*_p_^2^ =.008). Based on PTSD literature indicating differences for CS−, we performed independent samples *t*-tests comparing groups within each CS separate. FPS did not differ by group to either CS+ or CS− based on an independent samples *t*-test, *t*(69) = −1.29, *p* = .202 and *t*(69) = −1.25, *p* = .108 respectively. There were no other significant unique effects of sex or age on FPS at the end of fear conditioning.

### 3.4 | Fear extinction

**FIGURE 5:**
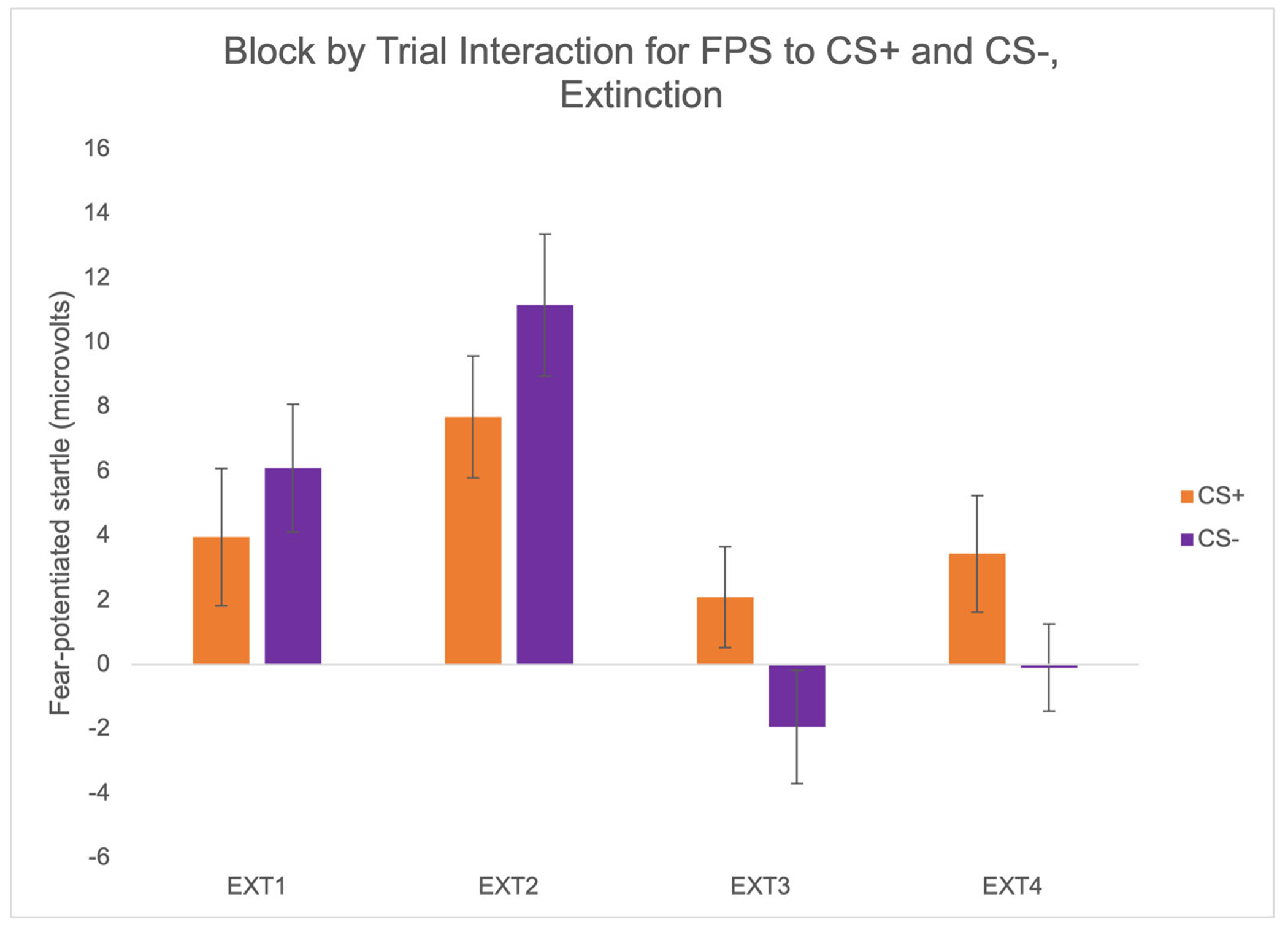
Psychophysiological responses over the course of fear extinction indicate diminished responses by the end of extinction. Error bars reflect standard error of the mean.

A 4 × 2 RMANOVA was used to determine whether there were significant effects of block (EXT1, EXT2, EXT3, and EXT4), trial type (FPS to nonreinforced threat cue CS+ and FPS to safety cue CS−), and block by trial type during fear extinction. There was a significant effect of extinction block, *F*(3,210) = 8.54, *p* < .001, *η*_p_^2^ = .11, and an interaction effect of block by trial type, *F*(2.56,179.33) = 2.93, *p* = .043, *η*_p_^2^ = .04 (Figure 5). The significant linear effect of block indicated a reduction in startle responses over the course of extinction, *F*(1,70) = 7.49, *p* = .008, *η*_p_^2^ = .10. With regard to extinction within each trial type, there was a significant reduction to the CS+ (*F*(3,210) = 9.03, *p* < .001, *η*_p_^2^ = .11) and the CS− (*F*(3,210) = 21.434, *p* < .001, *η*_p_^2^ = .23) over the four blocks of extinction. There were no differences in FPS to CS+ compared to CS− at the end of extinction (EXT4). There were no differences based on age, sex, or trauma exposure.

### 3.5 | Fear extinction and PTSD

**FIGURE 6:**
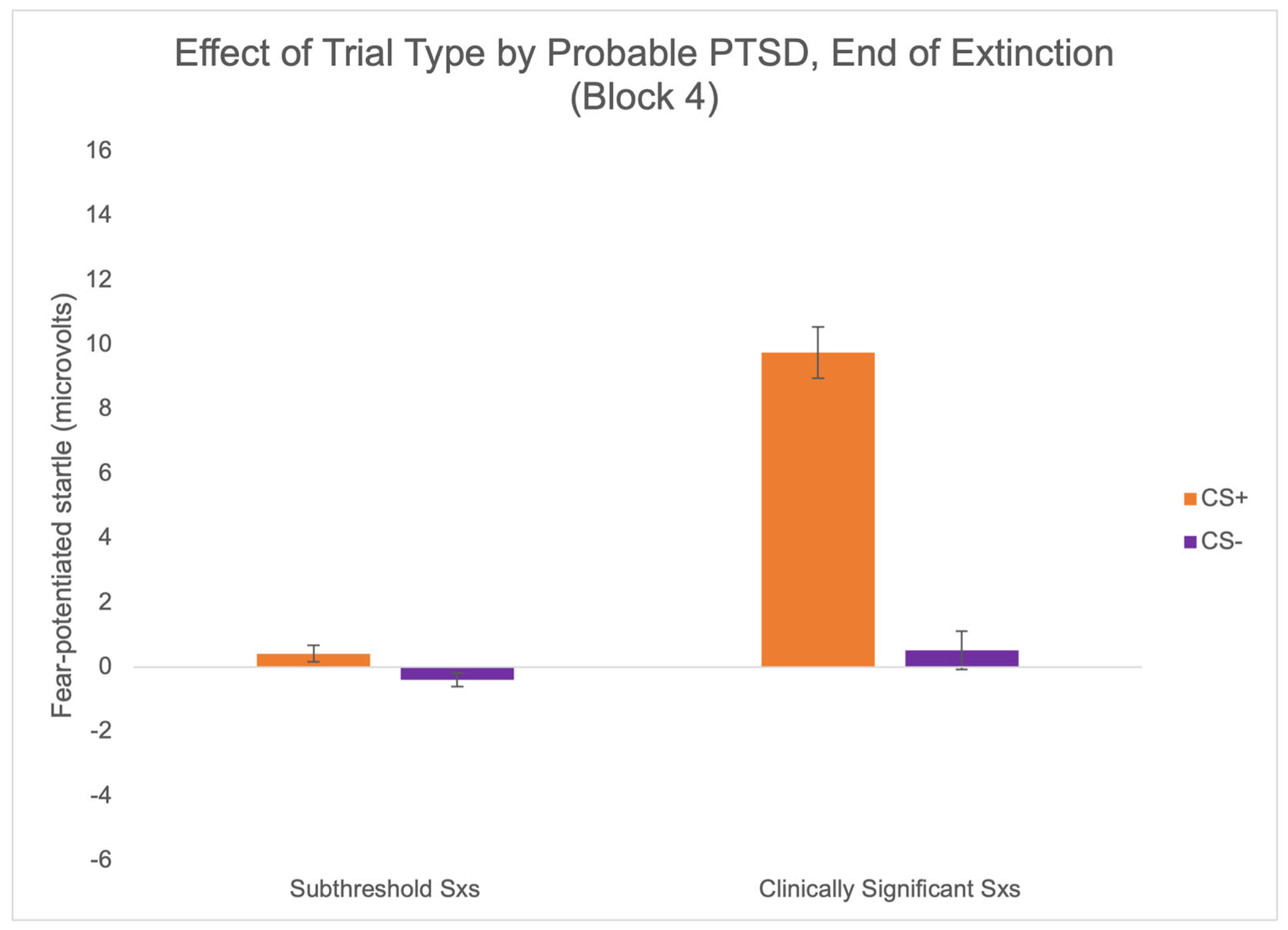
PTSD is associated with fear extinction. Comparison of FPS to threat and safety cues by probable PTSD at the end of extinction learning.

There was a significant interaction effect of trial type at the end of extinction (EXT4) by PTSD status, *F*(1,69) = 4.02, *p* = .049, *η*_p_^2^ = .06 (Figure 6). FPS to CS+ was significantly greater in the PTSD+ group compared to the PTSD− group, *F*(1,69) = 6.25, *p* = .015, *η*^2^ = .08, whereas FPS to CS− did not differ, *F*(1,69) =.09, *p* =.754, *η*^2^ = 001. FPS to CS+ at the end of extinction was not significantly associated with age, nor trauma exposure for the overall sample.

## 4 | DISCUSSION

The present study is, to our knowledge, the first to measure FPS in refugee youth and to measure extinction learning using the FPS paradigm in youth with and without probable PTSD based on self-reported PTSD symptom cutoff scores. We found unique effects of trauma exposure but not PTSD on fear conditioning—there was no group (PTSD+/−) by trial effect on FPS for the last block of fear conditioning (ACQ3), suggesting that PTSD. We had initially hypothesized that FPS at the end of fear conditioning would be associated with post-traumatic stress symptoms; however, our data led us to reject this hypothesis. Previous studies have found the greatest deficits in fear conditioning in individuals with the highest symptoms (Jovanovic et al., 2009; Norrholm et al., 2011). In this sample of youth, symptom severity may not have been high enough to yield discernable differences in fear conditioning. Rather, effects of PTSD emerged at the end of extinction learning: youth who screened positive for PTSD showed a deficit in extinguishing FPS to the threat cue. We found that impaired extinction learning and persistent fear potentiation of startle at the end of extinction occurs in youth with PTSD compared to those without. In accordance with the body of literature from adults, here too we see PTSD as a deficit in extinction learning, rather than dysregulated fear conditioning (Milad & Quirk, 2012; Milad et al., 2006; Milad et al., 2014; Norrholm et al., 2011). This study is the first to look at extinction learning in youth with and without PTSD. These results support the use of trauma-informed cognitive behavioral therapy in youth with PTSD, and the application of exposure therapy (Huang et al., 2022). These gold-standard treatments have been developed in adults with PTSD based on the phenomena that persons with PTSD fail to extinguish learned fear (Maples-Keller et al., 2022), and as our results corroborate this in youth, youth may also benefit from such treatment approaches. Alternatively, impaired extinction learning may be a reason exposure therapy might not work as well in a subgroup of individuals (Imel et al., 2013). In this case, emotion regulation-based methods such as cognitive processing therapy (Resick et al., 2016) and others may be appropriate. Given that our diagnoses of PTSD were based on clinical thresholds from self-report measures, this finding should be replicated in youth who screen positive for PTSD based on clinical interviews as well.

Our data also point toward greater threat reactivity in youth with higher trauma exposure and corroborate findings using other measures of threat reactivity in youth (Grasser et al., 2022). FPS to both threat and safety cues was positively correlated with trauma exposure, and discrimination was better in the higher trauma group, indicative of greater threat reactivity. While our data do not allow us to make causal conclusions, we may hypothesize that increasing trauma load contributes to greater physiological arousal in response to threat. However, these findings did not remain significant after controlling for age, and individuals of younger ages may show lack of discrimination between stimuli. This may be why the higher exposure group, which was also older, showed better discrimination. Given that age and trauma exposure are highly correlated, differentiating the unique effects of each will require larger samples with a nontrauma-exposed control group.

A limitation of this study is the use of self-report measures. Even in cases where data collection environments that establish trust with participants and engage members of the community in all stages of the process, there may still be limitations in terms of reporting, namely on self-report questionnaires. Participants may either over or under report symptoms. However, self-report questionnaires are beneficial in that they reduce time and burden on both participants and researchers compared to clinical interviews, and self-report questionnaires may be more readily adapted in a variety of integrative settings, including primary care clinics and resettlement agencies. Additionally, previous research has shown concordance between self- and clinician-administered measures of PTSD (Lenz & Williams, 2014; Lunney et al., 2014), indicating the accuracy and utility of such self-report measures for research. While large variation in biological data may limit generalizability of findings and require larger samples, our sample is a relatively demographically homogenous cohort with shared ancestry and similar experiences in trauma exposure, migration, and the resettlement process.

## 5 | CONCLUSION

There have been several advances in understanding the neurophysiological effects of trauma in recent decades. However, the vast majority of these studies are in Western, educated, industrialized, rich, and democratic (WEIRD) populations (Henrich et al, 2010a; Henrich et al., 2010b) and adults. On the other hand, research on trauma and stress among persons resettled as refugees have primarily focused on epidemiology. The present research was novel in its inquiry of FPS as a biological indicator of trauma-related psychopathology in MENA youth resettled from Syria. While causal conclusions cannot be made from this dataset and the corresponding analyses, we may hypothesize that continuous trauma exposure has contributed to a more reactive physiological state. We provided the first evidence in refugees that trauma exposure is associated with exaggerated fear-potentiated startle responses, and we corroborated data from adults that youth with PTSD show deficits in fear extinction. PTSD was associated with maintaining elevated responses to the threat cue at the end of extinction—providing further evidence to support the notion of PTSD as a disorder of fear extinction, rather than fear learning. Studying psychophysiological measures related to trauma-related psychopathology will advance knowledge and utility of potential treatment targets and could also be used as objective measures to evaluate such treatments. Physiological indicators could potentially provide objective and cost-effective measures (which would also overcome language barriers) to direct resource allocation and patient-centered care, especially for youth in vulnerable developmental states.

## Data Availability

Data and scripts for all analyses can be made available upon request to the corresponding author.
